# A review on the potential use of eubiotics in non-chicken poultry species

**DOI:** 10.1007/s11250-025-04466-9

**Published:** 2025-05-08

**Authors:** Caven M. Mnisi, Felix M. Njeri, Anderson N. Maina, Paul K. Waliaula, Veronica Cheng, Indibabale Kumalo, Chidozie F. Egbu, Elijah G. Kiarie

**Affiliations:** 1 Food Security and Safety Niche Area, Faculty of Natural and Agricultural Science, North-West University, Private Bag x2046, Mmabatho, 2735 South Africa; 2 Department of Animal Biosciences, University of Guelph, Guelph, ON N1G 2 W1 Canada

**Keywords:** Feed additives, Food safety, Gut health, Performance, Poultry

## Abstract

As the demand for poultry products increases, safe nutritional measures should be implemented to ensure successful diversification of the poultry industry with non-chicken poultry (NCP) species such as quail, turkey, ostrich, waterfowls, and guinea fowls. Thus, this review focuses on the current and future utility of eubiotics in NCP species by outlining the challenges and potential benefits that are associated with their utilization. Eubiotics are a group of feed additives, including probiotics, prebiotics, synbiotics, organic acids, and essential oils, that are safe and exhibit antimicrobial and immunomodulatory activities, prudent in an era where multi-drug antimicrobial resistance poses a grave threat to human health. Using eubiotics, separately or in combination, in NCP diets could enhance gut health, immune responses, growth performance, and product quality. However, their mechanisms of action are not fully understood, and their synergistic effects are not clearly outlined especially for NCP species. Moreover, inconsistent results have been reported, possibly due to various sources, application methods, production systems, bird types, and variations in rearing sites (macro- and micro-climatic conditions). We postulate that their extensive adoption in diets of NCP species could, in the future, deliver safe, efficient, and sustainable poultry production systems. We conclude that correct application methods, optimal dosages, and understanding of their synergistic actions could ensure alternative poultry systems that would contribute significantly to global food safety and nutrition security.

## Introduction

The poultry industry remains a cornerstone of global livestock production, providing a vital source of protein, income, and economic stability. While conventional chicken strains dominate commercial production (Vaarst et al. 2015; Mottet and Tempio 2017), non-chicken poultry species such turkeys, ducks, geese, quail, guinea fowl, and ostriches contribute significantly to food and nutrition security, rural livelihoods, and niche markets (Kokoszyński 2017; Vieira-Pires et al. 2021; Needham and Hoffman 2022). Additionally, the avian pet industry for non-consumable non-chicken poultry species such as parrots, canaries, pea fowl, finches, and other ornamental species, has gained substantial economic and cultural significance, driven by increasing demand for companion animals and conservation breeding programs.

Eubiotics, comprising probiotics, prebiotics, synbiotics, essential oils, and organic acids, are gaining recognition in poultry nutrition, demonstrating their potential to enhance performance indices and poultry product quality (Iebba et al. 2016; Oviedo-Rondón 2019; Anee et al. 2021). Probiotics, also known as direct-fed microbials, are a subset of eubiotics that contribute to improved gut health, nutrient absorption, and immune responses (Abd El-Hack et al. 2020; Jeni et al. 2021; Al-Hoshani et al. 2023), while prebiotics serve as substrates that support the growth of beneficial bacteria (Davani-Davari et al. 2019). Synbiotics, combining prebiotics and probiotics, further enhance the proliferation and survival of beneficial microbes (Jha et al. 2020; Maina et al. 2024). Additionally, organic acids are used as acidifiers that modulate gut pH and control pathogenic proliferation (Dittoe et al. 2018), while essential oils are extract products from natural plants that contain bioactive compounds with antimicrobial effects (Dhifi et al. 2016; Adaszyńska-Skwirzyńska and Szczerbińska 2017).

With the increasing demand for affordable animal protein, non-chicken poultry (NCP) species such as turkey, ducks, quail, ostrich, and guinea fowl are also gaining prominence (Huang et al. 2012; Fouad et al. 2018). The commercial emergence of these birds necessitates re-evaluating production and health management strategies (Nhung et al. 2017) and thus, eubiotics are emerging as sustainable and safe additives for use in NCP production. The impact of eubiotics on poultry is complex and influenced by factors such as strain selection, dosage, and management practices (Mehdi et al. 2018; Waliaula et al. 2024). Their application in NCP species, with unique physiological and metabolic characteristics, requires further investigation (Jha et al. 2020). The role of eubiotics in gut microbiota, crucial for performance and health, is an essential research area, especially for non-chicken birds where their influence remains less understood (Mehmood et al. 2023; Waliaula et al. 2024). Therefore, this review explores the potential of eubiotics in enhancing the production efficiency of NCP species. Additionally, the review discusses the potential of eubiotics in safeguarding NCP health and performance under different production conditions.

## Overview of non-chicken species

### Ostrich

The global demand for ostrich products, including meat, leather, and feathers, continues to rise, driven by interest in exotic meats, luxury goods, and sustainable livestock alternatives (Kokoszyński 2017). Valued for its low fat and cholesterol content, ostrich meat appeals to health-conscious consumers, contributing to the species’ growing market presence. South Africa leads global production, housing 75% of farmed ostriches and projecting 150,000 birds for slaughter in 2024/25, a 7% increase driven by improved returns on feathers and leather, stable meat prices, and lower feed costs. Beyond South Africa, regions like the United States, Europe, and Asia are expanding ostrich farming to meet rising demand for eco-friendly, nutritious meat, though the market remains niche (Needham and Hoffman 2022). The sector’s profitability extends to leather and feathers, particularly in luxury and industrial markets. However, challenges like fluctuating demand, disease outbreaks, regulatory barriers, and competition from emerging producers, especially in China and the Middle East, continue to shape the industry’s future.

### Quail

Although the demand for quail products is expanding, official figures on quail production are insufficient due to poor recording procedures on small-scale farms, which generate most of the meat and eggs (El Sabry et al. 2022). Approximately 1.4 billion broiler quail are annually raised worldwide for both egg and meat production (Katerynych and Pan’kova 2020). Additionally, Katerynych and Pan’kova (2020) noted a prospective market for quail meat, with consumption increasing annually by 5–10%. Available data suggest that more than 80% of the quail are produced in tropical and subtropical countries including China, Indonesia, India, Japan, Brazil, and Mexico. In Africa, data on the quail egg and meat market is generally limited and many countries lack statistics on quail farming and market size for eggs or meat. However, two large-scale companies partly serve the Egyptian market with quail products. Mnisi et al. (2021) also noted that South Africa has over six large and several hundred small-scale registered quail producers.

### Guinea fowl

Guinea fowls are native to Africa and are commonly raised for meat and eggs in many African countries (Houndonougbo et al. 2017). However, detailed production statistics may not always be readily available or regularly updated. The population of guinea fowl in Nigeria is over 50 million and is widely distributed in the savanna areas of the country. Guinea fowl production in Africa is often done at a subsistence level, with limited production inputs. Guinea fowl production also occurs in some European countries, with France being one of the notable producers. France has a tradition of rearing guinea fowl for both meat and eggs. Guinea fowl production in North America, particularly in the United States, are small-scale producers and backyard farmers often reared for pest control and their unique-tasting meat. Some countries in Asia, such as India, have small-scale guinea fowl production mainly for personal consumption or local markets. However, in Australia and New Zealand, they are often kept for ornamental purposes or as hobby birds rather than for commercial production (Houndonougbo et al. 2017).

### Turkey

The turkey sub-sector represents one of the major pillars of the poultry industry (Kokoszyński 2017), with turkey meat being the second most consumed poultry meat worldwide with an average annual consumption of 4.0 and 7.3 kg/per capita in Europe and the United States, respectively (Kokoszyński 2017; Zampiga et al. 2020). The highest production of turkey meat is in America (55.3%), followed by Europe (36.2%), Africa (4.9%), Asia (3.1%), and the lowest is Oceania with 0.4%. The world yield of turkey meat in 2022 was over 5 million tons (Vieira-Pires et al. 2021).

### Ducks

Ducks play a vital role in global poultry production, contributing to meat, eggs, and cultural cuisines, particularly in Asia, which holds 90.9% of the world’s duck population. Between 1961 and 2019, global duck numbers expanded sixfold from 193.4 million to 1.18 billion birds (Jalaludeen et al. 2022), driving a 2022 yield of over 6 million tons of meat (FAOSTAT 2024). Beyond their economic value, ducks hold cultural significance in many regions, featuring prominently in traditional dishes and farming systems, particularly in integrated rice-duck agriculture, which supports sustainable food production.

### Geese

Geese represent a significant yet specialized segment of NCP production, valued for their meat, eggs, feathers, and weed control capabilities in agricultural systems (Kozák 2021; Bao et al. 2022; Salamon 2025). Adapted to foraging-based production, geese require minimal inputs compared to other poultry species, making them well-suited for extensive and semi-intensive farming. Global goose production is dominated by China, which accounts for over 95% of the world’s output, with annual meat yields exceeding 4.5 million tons (FAOSTAT 2024). Other notable producers include Egypt, Poland, and Hungary, where geese are raised for high-value products such as foie gras, down feathers, and traditional cuisine (Kozák, 2021; Wei and Han 2025). Due to their resilience, long lifespan, and ability to utilize fibrous plant material efficiently, geese contribute to sustainable poultry systems while supporting rural economies and niche markets (Kuźniacka et al. 2019; Lin et al. 2023).

### Ornamental non-chicken species

Ornamental species, including peafowls, parrots, canaries, and finches, hold an important niche within poultry production, driven by their economic, ecological, and cultural value (Chomel et al. 2007). While not traditionally part of food systems, these birds contribute significantly to the pet trade, avian tourism, and conservation breeding programs. Peafowls, with their striking plumage, are prized in ornamental aviaries and estates, while parrots, canaries, and finches are popular companion birds, fueling a growing global pet industry (Chomel et al. 2007; Enferadi et al. 2024). Beyond aesthetics, these species play a role in genetic conservation, preserving rare and endangered avian breeds through captive breeding programs.

### Impact of eubiotics on performance, gut health, and product quality in NCP species

**Fig. 1:**
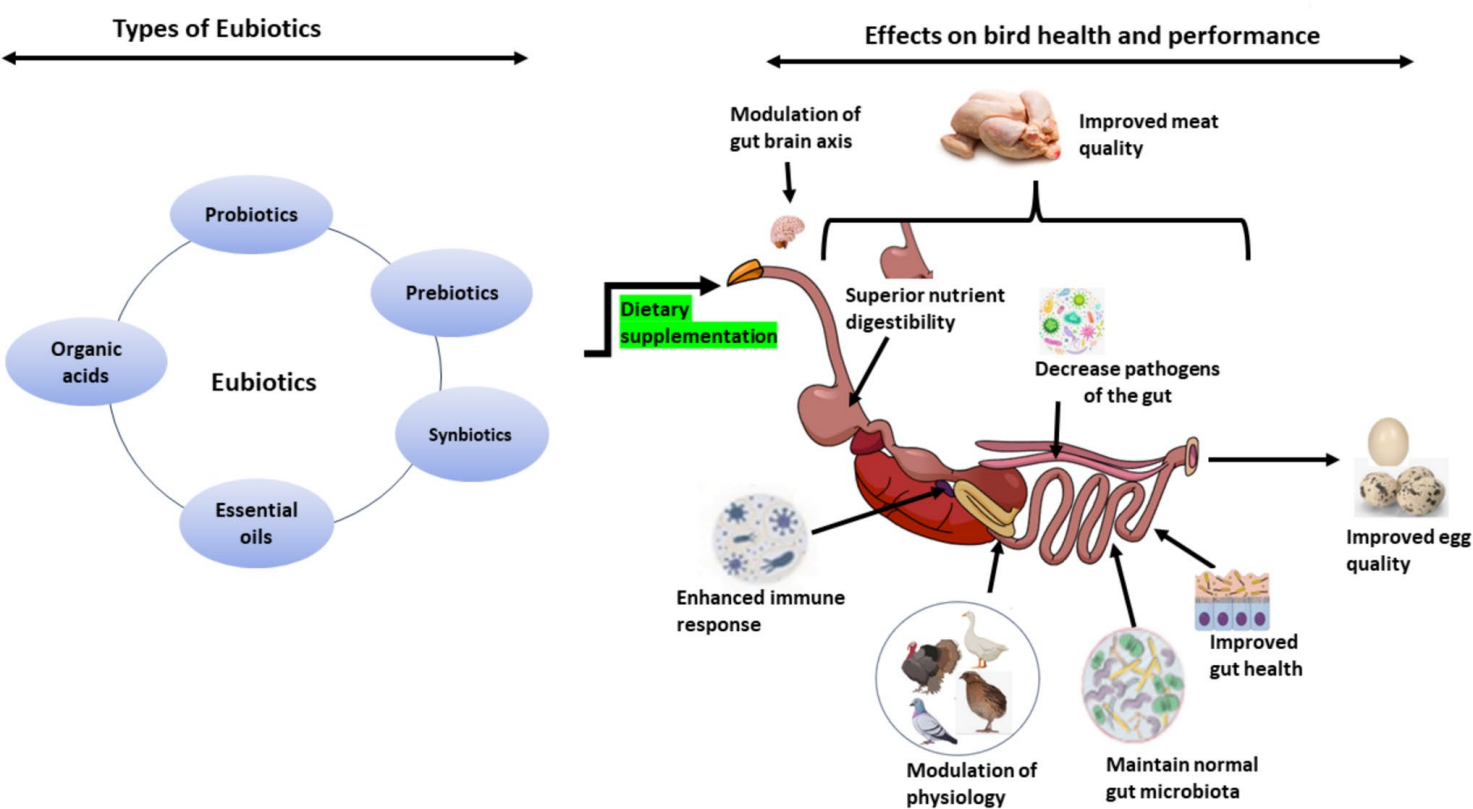
The anticipated benefits of eubiotics in non-conventional poultry birds

Over the years, researchers worldwide have prioritized investigating critical factors such as performance metrics, gut health measurements, and meat quality attributes to ensure the efficiency, sustainability, and economic viability of poultry enterprises (Muneer et al. 2022; De Cloet et al. 2023; Dablool et al. 2024; Maina et al. 2025), as depicted in Fig. 1. In response to increasing consumer awareness and demand for safe, antibiotic-free, high-quality eggs and meat, the industry has shifted inevitably towards exploring safe and natural alternatives like eubiotics (Bean-Hodgins and Kiarie 2021; Maina et al. 2022; Dablool et al. 2024). Some studies have reported on the utilization of eubiotics in waterfowl and quail production (as summarized in Table 1) but there have been minimal reports on turkeys, guinea fowl, geese and ostriches and almost no studies on ornamental NCP species such as peafowls, parrots, canaries, and finches among others. In this context, this section reviews the reported impact of eubiotics in NCP species currently of commercial interest.

**Table 1:** Summarized effects of different eubiotic additives on performance and health parameters of NCP species

Eubiotic additive type	Species	Administration route	Inclusion rate	Findings	References
Probiotics	Meat-type Japanese quail	Oral, in-feed	*Bacillus toyonensis* (1 mL/kg diet, 5 × 10^8 CFU/mL) and *Bifidobacterium bifidum* (0.5 mL/kg diet, 1 × 10^8 CFU/mL)	↔ Feed intake ↔ FCR ↓ Total coliform count ↓ Meat color values ↓ Proteolysis ↓ Cooking loss ↓ Thiobarbituric values	(Abou-Kassem et al. 2021)
Dietary probiotics + humic acid	Meat-type Japanese quail	Oral, in-feed	5–10 g/kg	↑ Phosphorus in probiotic females ↓ HDL cholesterol ↔ Meat quality	(Kalafova et al. 2018)
Compound probiotics	Shaoxing ducks	Oral, in-feed	0.15% compound probiotics (MixP)	↑ Cecal microbiome and metabolome	(Sun et al. 2022)
Encapsulated essential oils	Cherry Valley meat ducks	Oral, in-feed	500 and 1000 mg/kg	↑ Growth performance ↑ Intestinal health ↑ Egg production	(Bao et al. 2023)
Herbal essential oil	Layer quail	Oral, in-feed	24 mg/kg feed in place of antibiotic (avilamycin)	↑ Egg production ↑ FCR	(Çabuk et al. 2014)
Organic acids	Quail	Oral, in-feed	Humic acids at 0.5% and 1.0%	↔ Final body weight ↔ Carcass traits ↓ Blood cholesterol levels	(Zigo et al. 2020)
Probiotics, *Bacillus subtilis*	Pekin ducks	Oral, in-feed	1 g/kg	↓ Stress fear response ↓ Corticosterone ↓ Heat shock protein	(Mitin et al. 2022)
Essential oils, probiotics and prebiotics	Japanese quail breeders	Oral, in-feed	EOs (250 mg/kg), probiotics (150 mg/kg), mannan-oligosaccharides (2 g/kg)	↔ Egg production ↔ Hatchability ↑ Intestinal histopathology ↑ Microbiota activity ↓ Serum cholesterol	(Hajiaghapour and Rezaeipour 2018)
Probiotics	Tom turkeys	Oral, in-feed	*Bacillus subtilis*: Lower doses: 1E + 08 cfu/kg and 2E + 08 cfu/kg Higher dose: 1E + 09 cfu/kg *Bifidobacterium longum* PCB133	↑ Growth with lower doses of *Bacillus subtilis* ↑ Nutrient retention and gut health with higher doses of *Bacillus subtilis* ↔ Growth with *Bifidobacterium longum* PCB133 ↔ Immune response with *Bifidobacterium longum* PCB133 ↔ Vaccination efficiency against Newcastle disease with *Bifidobacterium longum* PCB133	(Mohammadigheisar et al. 2019)
Probiotics	Ostrich chicks	Oral, in-feed	T2: 0.04% Bioplus 2B T3: 0.09% Primalac T4: 0.1% Thepax T5: 0.03% Protexin	↑ Body weight gain ↑ Total cholesterol	(Karimi-Kivi et al. 2015)
Benzoic acid and essential oil compounds	Turkey Poults	Oral, in-feed	Benzoic acid 300 and 1,000 mg/kg, (thymol (≥ 10%), eugenol (≥ 0.5%), piperine (≥ 0.05%)	↑ Growth performance ↑ Lactic acid bacteria ↓ Coliform bacteria ↓ Cecal buffering capacity ↓ pH	(Giannenas et al. 2014)
Fumaric acid	Japanese quail	Oral, in-feed	5, 10, 15, and 20 g/kg	↑ Growth performance ↑ Nutrient retention ↑ Blood chemistry ↓ Coliform *E. coli* count ↓ *Salmonella* count	(Bao et al. 2023)
Citric acid	Japanese quail chicks	Oral, in-feed	5, 10, 15, and 20 g/kg	↑ Improved growth performance, ↑ Gut health, and ↑ Immune response	(Fikry et al. 2021a, b)
Probiotics	Guinea fowls	Oral, in-water	1.5 ml/L	↑ Weight gain ↓ Feed consumption ↔ Blood and serum biochemical properties	(Sarfo et al. 2018)
Organic acids a competitive exclusion product probiotic	Turkeys	Oral, in-feed	Organic acids (2 g/kg) and a competitive exclusion product (10^9 cfu/kg)	↓ Early growth ↓ Feed intake ↑Volatile fatty acids in cecum ↔ Intestinal morphology with	(Milbradt et al. 2014)
*Bacillus subtillis* probiotic	Geese	Oral, in-feed	30 and 60 ppm	↑ Final body weight & daily gain (60 ppm) ↑ Feed efficiency (30 & 60 ppm) ↑ Ileum villi height (30 & 60 ppm) ↑ Digestive enzyme activity (30 & 60 ppm) ↑ Nutrient digestibility (60 ppm) ↑ Beneficial gut bacteria (60 ppm)	(Li et al. 2025)
*Bacillus subtillis* probiotic	Geese	Oral, in-feed	5 × 10^9^ CFU/kg BS	↑ Hatching rate, eggshell thickness, antioxidant capacity, intestinal structure, beneficial gut microbiota	(Wang et al. 2020; Fan et al. 2022)

Key: ↑, Improved; ↓, Decreased; ↔, No effect

### The use of eubiotics in quail

Quail, recognized for their small size, rapid growth rates, flavorful meat, nutraceutical eggs, and exceptional laying and growth performances (Mahlake et al. 2021), have emerged as key contributors to the poultry industry over the past decade. Originally utilized as model animals in avian research, quail production for human consumption has increasingly gained traction. Studies on probiotic supplementation in quail, including strains such as *Bacillus toyonensis* (1 mL/kg diet, 5 × 10^8^ CFU/mL) and *Bifidobacterium bifidum* (0.5 mL/kg diet, 1 × 10^8^ CFU/mL), administered either individually or in combination, showed no significant effects on feed intake or feed conversion ratio (FCR) (Abou-Kassem et al. 2021). However, Japanese quail supplemented with these probiotics exhibited reduced total coliform counts, improved meat color stability, lower proteolysis, decreased cooking loss, and reduced thiobarbituric acid values (indicating reduced lipid oxidation). In a separate study, a multi-strain probiotic (1 × 10^9^ CFU/g) enhanced small intestine morphology (increased villus height and intestinal length), serum biochemical markers (uric acid, glucose, and total protein levels), and nutrient absorption (Seifi et al. 2017) Notably, this probiotic did not influence immune response parameters. Kalafova et al. (2018) explored the combined effects of probiotics (1 g/kg feed) and humic acids (3 g/kg feed), revealing sex-specific outcomes: serum calcium levels increased in females, while serum phosphorus levels rose only in probiotic-treated females. Both additives reduced HDL cholesterol in females, with pronounced sex-based differences in LDL/HDL cholesterol profiles. Additionally, meat pH varied significantly across muscle types 24 h post-slaughter, underscoring dose-dependent physiological impacts of these supplements in Japanese quail.

Supplementation with a 150 mg/kg multi-strain probiotic, including *Aspergillus oryza*, *L*. *delbrueckii* subsp. *Bulgaricus*, *L*. *acidophilu*s, *L*. *rhamnosus*, *Candida pintolopesii*, *Bifidobacterium bifidum*, *Streptococcus salivarius* subsp. *Thermophilus*, *E*. *faecium*, and *L*. *plantarum*, enhanced growth performance, organ weights, and the immune system in Japanese quail-fed diets contaminated with aflatoxin B1 (Bagherzadeh Kasmani and Mehri 2015). Tekce et al. (2020) reported improved body weight gain (BWG), FCR, and feed intake (FI) upon supplementation of *Lactobacillus reuteri* probiotic (4 × 10^10^ CFU/g) from day 7–35 in heat-stressed Japanese quail. Similarly, 0.4 mg/kg of *Paenibacillus polymyxa* probiotic (1 × 10^6^ CFU/g) improved productive performance and intestinal health by enhancing antioxidative status, immune response, and beneficial bacterial populations while reducing *E. coli* count in Japanese quail (Alagawany et al. 2021a, b). Additionally, Soomro et al. (2019) demonstrated that probiotic supplementation enhanced FCR and reduced mortality rates in meat quail. In a study by Nour et al. (2021) while supplementing laying Japanese quail diets with *Bacillus toyonensis* (B1) and *Bifidobacterium bifidum* (B2) at varying doses (0.10% B1 alone, 0.10% B2 alone, and a combination of 0.10% B1 with 0.05% B2) resulted in enhanced egg production, egg weight, feed efficiency, fertility, hatchability, and favourable blood biochemical profiles. These findings underscore the potential of B1 and B2 probiotic strains to significantly improve the overall performance and health of laying Japanese quail.

Essential oils have been reported in quail studies. Alagawany et al. (2021b) found that the dietary supplementation of lemongrass essential oil (LGEO) in growing quail diets demonstrated positive effects across various parameters. Specifically, supplementation with LGEO at levels of 150, 300, and 450 mg/kg improved body weight, feed conversion ratio, plasma lipid profile, immunity markers, antioxidant indices, and reduced intestinal pathogens, suggesting that LGEO has the potential to enhance the overall health and performance of growing quail. Khalifah et al. (2021) also found that supplementing LGEO at 0.4 g/kg in growing quail diets positively impacted growth performance, carcass traits, meat quality, and blood characteristics. The LGEO supplementation significantly improved parameters such as water holding capacity and pH values of thigh meat while simultaneously enhancing antioxidant capacity, reducing lipid profiles and markers of liver function, and promoting the growth of beneficial bacteria in the caecum, suggesting its potential as a multifaceted additive for overall health and performance in quail nutrition. In addition, Gumus et al. (2017) found that the inclusion of thyme essential oil (TEO) in quail diets, particularly at the highest level (TEO3, 450 mg/kg), led to increased body weight and daily weight gain. Additionally, TEO supplementation positively influenced serum parameters, including reduced creatinine and low-density lipoprotein (LDL) levels, elevated serum magnesium levels, and notably enhanced antioxidant metabolism in both liver and serum, as evidenced by increased catalase (CAT), superoxide dismutase (SOD), and glutathione peroxidase (GSH-Px) activities, along with reduced lipid peroxidation levels (Gumus et al. 2017).

Dietary supplementation of ajwain essential oil (AEO) at 250 mg/kg positively impacted quail breeders’ feed conversion ratio, indicating improved feed efficiency. Furthermore, AEO supplementation led to a decrease in blood serum cholesterol levels and an increase in the number of Lactobacilli in the ileocecal region, demonstrating its potential benefits on lipid metabolism and gut microbiota in quail breeders (Hajiaghapour and Rezaeipour 2018). Dehghani et al. (2018) reported that dietary supplementation of thyme and savoury essential oils at levels of 400 ppm resulted in a significant improvement in feed conversion ratio by reducing feed intake (FI) without affecting the body weight gain (BWG). Additionally, both essential oils positively influenced intestinal morphology, with increased villi height and decreased crypt depth, indicating their potential to enhance nutrient absorption in quail. Laying quail supplemented with an essential oil mixture (EOM, 24 mg/kg feed) consisting of oregano, laurel leaf, sage leaf, myrtle leaf, fennel seeds, and citrus peel, or an antibiotic (avilamycin, 10 mg/kg feed), showed increased egg production compared to a control diet (Çabuk et al. 2014). Both EOM and antibiotic treatments had similar effects on egg production, with no differences on egg weight or feed intake, but significantly improved feed conversion ratios, indicating the beneficial effects of EOM as a dietary supplement on egg production and feed efficiency (Çabuk et al. 2014).

Further, the dietary supplementation of 2 g/kg TEO in Japanese quail increased final body weight, improved feed conversion ratio, and significantly enhanced serum biochemistry by increasing HDL-cholesterol levels. While thyme exhibited negligible effects on performance criteria, its positive impact on serum parameters suggests its potential as a beneficial additive in quail nutrition, offering improvements in specific health-related aspects (Kheiri et al. 2018). The dietary supplementation of a phytogenic feed additive (PFA) at increasing levels (125, 250, 500, and 1000 mg/kg) in the diets of female Japanese quail breeders led to notable improvements in various parameters (Safavipour et al. 2022). These included increased egg weight, feed efficiency, shell-breaking strength, calcium content, specific gravity, Haugh unit, and percentages of fertile eggs, along with enhanced nutrient digestibility, enzyme activities, gut microbiota balance, villus morphology, and immune-related indicators, collectively indicating that PFA has the potential to enhance gut health, nutrient utilization, and overall productivity and fertility in quails. A study utilizing *Lippia gracilis* Schauer essential oil (LGSEO) demonstrated its potential as a growth promoter for Japanese quail. When included at a dose of 400 mg/kg in the diet, LGSEO showed benefits such as improved feed conversion, restricted *Escherichia coli* growth, and modulation of intestinal gene expression, highlighting its effectiveness in enhancing performance and creating a favourable intestinal environment for the quail (Rocha et al. 2020). Cold-pressed clove oil (CCPO) in the dietary supplementation at a 1.5 mL/kg level demonstrated positive effects on growing Japanese quails’ growth performance and health (Hussein et al. 2019). Quail on the 1.5 mL CCPO/kg diet group showed improved live body weight, daily weight gain, feed conversion ratio, positive changes in antioxidant enzyme activities, lipid profile, and reduced glutathione concentrations.

Additionally, urea, creatinine, malondialdehyde, 8-hydroxy-2΄-deoxyguanosine, and protein carbonyl decreased. In contrast, serum levels of insulin-like growth factor-1, insulin, growth hormone, and thyroxine increased, indicating the potential of CCPO to enhance the overall quality of health (Hussein et al. 2019). Additionally, Türk et al. (2015) found that heat stress (HS) negatively impacted the reproductive parameters of developing male Japanese quail, including decreased body weight, spermatid and testicular sperm numbers, and alterations in apoptotic and antiapoptotic markers. However, supplementation of cinnamon bark oil (CBO) at doses of 250 and 500 ppm significantly mitigated the adverse effects of HS, showing improvements in testicular lipid peroxidation, sperm numbers, antiapoptotic marker density, and androgenic receptor immunopositivity, highlighting the potential protective role of CBO against HS-induced testicular damage in quail. Lastly, dietary supplementation with red pepper oil (RPO) at 0.8 g/kg significantly improved the growth performance of growing quail, showing increases in live body weight, body weight gain, and feed conversion ratio (Reda et al. 2019). Additionally, RPO supplementation at this level positively influenced antioxidant status, as reflected by higher activities of glutathione and catalase and decreased levels of intestinal pathogens, suggesting its potential as a beneficial additive to enhance the health and performance of Japanese quail (Reda et al. 2019).

Organic acid supplementation in quail diets has been investigated, although not extensively. For example, Zigo et al. (2020) reported that the supplementation of humic acids (HA) at levels of 0.5 and 1.0% in the diet of Japanese quail for 52 days did not affect the final body weight and carcass traits. However, the 1.0% HA supplementation demonstrated a positive impact by reducing blood cholesterol levels, suggesting HA’s potential role in producing quality meat with lower fat content in quails. Fumaric acid (FUA) dietary supplementation in Japanese quail chicks’ diets, particularly at 15 g/kg, significantly enhanced growth performance, nutrient digestibility, immune response, antioxidant status, and digestive enzyme activity (Reda et al. 2021). Additionally, FUA supplementation improved plasma lipid profiles and reduced cecal counts of harmful bacteria, indicating its positive effects on overall performance, health, and intestinal microbiota in Japanese quail chicks (Reda et al. 2021). Fikry et al. (2021a, b) found that the supplementation of citric acid (CA) in the diets of growing Japanese quail significantly improved growth performance, with higher live body weights and weight gain observed in CA-treated groups compared to the control.

Additionally, CA supplementation enhanced nutrient digestibility, digestive enzyme activities, and immune response, as indicated by increased albumin, globulin, and IgG levels, including CA, particularly at 10 g/kg, proved beneficial for overall performance and health in growing Japanese quail. Eubiotics have been used extensively in quail with inconsistent results on growth performance. Nonetheless, these additives appear essential in reducing the abundance of *E. coli* and *Salmonella* at the caecal level while increasing the number of beneficial microbes such as *Lactobacilli*.

### The use of eubiotics in turkey

Turkeys, prized for their superior muscle deposition compared to broiler chickens, have experienced surging global production demand (Kokoszyński 2017; Vieira-Pires et al. 2021). With the global turkey meat export market valued at USD 5.89 billion in 2021, optimizing feeding strategies for performance and product quality has become critical. Eubiotics, including probiotics, prebiotics, and essential oils, have emerged as promising dietary interventions in turkey nutrition.

Studies on probiotic supplementation in turkeys reveal dose-dependent outcomes. For instance, *Bacillus subtilis* at lower doses (1 × 10⁸ and 2 × 10⁸ CFU/kg) improved growth in Hybrid Converter Toms, while higher doses (1 × 10⁹ CFU/kg) enhanced nutrient retention and gut health (Mohammadigheisar et al. 2019). Conversely, *Bifidobacterium longum* PCB133 showed no benefits for growth, immune response, or Newcastle disease vaccination efficacy in young turkeys (Seifert et al. 2011). In contrast, Bacillus probiotic PHL-NP122 (10^6^ spores/g feed) increased body weight and reduced Salmonella prevalence (Wolfenden et al. 2011), while Primalac (1.5 kg/ton feed) decreased Clostridium perfringens colonization without affecting growth metrics (Rahimi et al. 2011). Further, Duff et al. (2020) assessed the impact of the synbiotic feed additive, containing *Lactobacillus reuteri*, *Enterococcus faecium*, *Bifidobacterium animalis*, *Pediococcus acidilactici*, and a fructo-oligosaccharide prebiotic, on turkey poults, which improved weight gain and reduced intestinal health issues in poults challenged with *Eimeria* and *Salmonella*, indicating its effectiveness in poultry health management.

Organic acid supplementation yields mixed results. A blend of organic acids (2 g/kg) and a competitive exclusion product (10⁹ CFU/kg) reduced early growth and feed intake but increased cecal volatile fatty acids, though intestinal morphology remained unaffected ((Milbradt et al. 2014). Protected forms of butyric acid, coated sodium butyrate (CSB) and butyric acid glycerides (BAG), enhanced feed conversion ratio (FCR), European Efficiency Index (EEI), duodenal villus height, and protein digestibility while reducing fecal *E. coli* and C. perfringens (Makowski et al. 2022). Both CSB and BAG elevated cecal butyric acid levels, highlighting their potential for gut health optimization.

Functional oils (0.15% cashew nutshell and castor oil blend) improved early weight gain (4.5% by week 12) and reduced meat oxidation, though effects diminished post-week 13 (Ferket et al. 2020). Monensin (at 66 ppm) outperformed functional oils, boosting weight by 10.5% and enhancing feed efficiency. Similarly, benzoic acid (300–1,000 mg/kg), thymol (30 mg/kg), and mixed essential oils (MEO, 30 mg/kg) increased growth rates, elevated beneficial lactic acid bacteria, reduced coliforms, and improved antioxidant status. Combining benzoic acid with MEO also lowered caecal pH and feed buffering capacity (Giannenas et al. (2014). While probiotics, prebiotics, and essential oils demonstrate potential to enhance turkey performance, gut health, and pathogen resistance, outcomes are highly dose- and strain-dependent. Organic acids and butyrate derivatives show promise but require further validation in commercial settings. Strategic selection and optimization of eubiotics formulations are essential to maximize their benefits in turkey production.

### The use of eubiotics in ducks

Waterfowl, primarily known as ducks, are commercially significant poultry worldwide, thriving in aquatic habitats for breeding (Biswas et al. 2019). While Asian countries are the highest producers of ducks, the per capita consumption is highest in Europe, with France on the frontline (Jalaludeen and Churchil 2022). To enhance their performance, immune response, and product quality, eubiotics have been incorporated into duck diets.

Probiotics have shown significant benefits in improving growth, health, and productivity in ducks. Khattab et al. (2021) found that supplementing white Pekin ducks with a probiotic blend (0.2 g/kg diet) containing *Lactobacillus acidophilus* and Lactobacillus casei significantly enhanced growth, intestinal health, immune function, and antioxidant activity, particularly in diets with higher crude protein (CP) levels (18%). Ducks on lower-protein (14%) diets without probiotics performed poorly; however, probiotic supplementation improved their outcomes, demonstrating its effectiveness in promoting growth and health, especially in low-CP diets. In laying ducks, probiotics have been linked to improved egg production and gut health. Li et al. (2011) reported that supplementing *Bacillus subtilis* (1 × 10^8^ CFU/kg) in the diet of Shaoxing ducks improved their egg-laying rate, positively altered egg composition, and enhanced blood biochemistry. It also significantly increased beneficial gut microflora, indicating overall positive effects on the health and productivity of laying ducks. Similarly, Cao et al. (2022) found that probiotics enhanced feed intake, egg production, body weight, and serum superoxide dismutase activity. However, dietary acidifiers, while increasing yolk weight and influencing calcium-binding and reproductive gene expressions, reduced serum antioxidant and immune capacity.

Probiotic supplementation has also been associated with beneficial shifts in gut microbiota and metabolism. For instance, Sun et al. (2022) observed that Shaoxing ducks supplemented with 0.15% compound probiotics (MixP) exhibited significant changes in their intestinal microflora and metabolic profile. These ducks had a higher abundance of *Bacteroidete*s and *Bacteroides* and lower levels of *Firmicute*s, *Oscillospira*, and *Desulfovibrio*. Additionally, MixP altered 71 metabolites and influenced key pathways such as vitamin B6 metabolism and protein digestion, suggesting improved cecal health. Improvements in immune response and disease resistance have also been noted with probiotic supplementation. Guo et al. (2016) reported that feeding *Bacillus subtilis*-supplemented diets to Cherry Valley ducks led to significantly higher body weight, better gut morphology, and enhanced immune organ weights. Additionally, these ducks exhibited increased pro-inflammatory factors and antiviral proteins at 28 days post-hatch. The survival rates of probiotic-fed ducks against *Escherichia coli* and novel duck reovirus were 43.3% and 100%, respectively, highlighting probiotics’ role in enhancing immune function and disease resistance. Certain probiotics may also influence cholesterol levels. Kismiati et al. (2022) found that supplementing Pengging ducks with synbiotics (containing inulin from gembili tubers and *Lactobacillus plantarum* Ina CC B76) significantly reduced egg yolk cholesterol, particularly at the 1.5 mL/100 g dose. However, this supplementation did not significantly affect egg production, egg quality, or hematological parameters. Meanwhile, dietary acidifiers (2–3 g/kg; containing benzoic, fumaric, phosphoric, and formic acids) and probiotics (*Bacillus subtilis* and *Clostridium butyricum*) have been shown to influence production, egg quality, and gene expression in Cherry Valley ducks (Cao et al. 2022).

Although research on essential oil supplementation in ducks is limited, available studies suggest potential benefits. Ge et al. (2023) reported that Muscovy ducks feeding a basal diet supplemented with 200 mg/kg essential oils (EO) had an improved final body weight, average daily gain, and feed conversion ratio over 56 days. This EO supplementation also enhanced antioxidant capacity, immune function, intestinal barrier function, and positively modulated gut microbiota, increasing beneficial short-chain fatty acid-producing bacteria and decreasing potential pathogenic bacteria. However, not all essential oils improve growth performance. Abouelezz et al. (2019) found that growing ducks supplemented with oregano essential oil (OEO, 150–300 mg/kg; 5% thymol, 65% carvacrol) and Enviva essential oil (EEO, 50–100 mg/kg; 4.5% cinnamaldehyde, 13.5% thymol) showed no significant differences in body weight, growth rate, feed intake, feed conversion ratio, or survivability. However, these essential oils reduced harmful cecal bacteria (*Coliforms* and *Enterobacteria*) without significantly affecting serum biochemical markers.

Ding et al. (2020) observed that oregano EO supplementation (100 mg/kg) improved feed intake, intestinal health, and antioxidant capacity in ducks, yielding effects comparable to antibiotic (500 mg/kg aureomycin) treatment. Ducks on EO diets exhibited increased villus height-to-crypt depth ratios, higher serum superoxide dismutase activity, and reduced malondialdehyde levels, suggesting antimicrobial potential. Similarly, Bao et al. (2023) found that Cherry Valley ducks supplemented with encapsulated essential oils (EOs) at 500 mg/kg (LEO) and 1000 mg/kg (HEO) had improved growth performance, with the HEO group showing higher average daily feed intake, gain, body weight, and a lower feed conversion ratio compared to the control and chlortetracycline (50 mg/kg) groups. These EO supplements also positively impacted intestinal health by increasing villus heights, enhancing barrier function gene expression, and improving cecal microbiota diversity and composition, demonstrating their beneficial effect on gut microbiota and overall intestinal health in ducks.

In dealing with abiotic stressors, Mitin et al. (2022) reported that the administration of probiotics in Pekin ducks after being crated for 4 h reduced stress, as indicated by corticosterone measurements, heat shock protein 70, creatine kinase, triglyceride levels, and the ratio of heterophils to lymphocytes. In the same study, including probiotics in the diet decreased fear-associated behaviours among birds subjected to crating. Although eubiotics show promise in enhancing growth, health, and stress resilience in ducks, further research is needed to fully understand their mechanisms and long-term effects.

### The use of eubiotics in ostriches

Globally, ostriches are the largest and heaviest flightless birds, and their farming has been increasingly adopted to enhance egg and meat production. However, research on the efficacy of eubiotics in ostrich farming remains limited. Despite the scarcity of studies, existing research suggests that eubiotics may be beneficial, particularly for young ostriches, which are vulnerable due to their immature immune systems. Nevertheless, the few that exist support using eubiotics in young ostriches, vulnerable due to their immature immunity. For instance, in a six-week study on ostrich chicks (Karimi-Kivi et al. 2015), four diets supplemented with different probiotics (T2: 0.04% Bioplus 2B; T3: 0.09% Primalac; T4: 0.1% Thepax; T5: 0.03% Protexin) were compared to an un-supplemented control diet. Chicks on the Bioplus 2B supplemented diet (T2) showed generally higher body weight gain than those on the control diet, and probiotic consumption influenced several haematological parameters, with T2 and T3 increasing total cholesterol compared to the control group (Karimi-Kivi et al. 2015).

Further evidence of probiotics’ benefits in ostriches comes from a study on farm-raised birds conducted by Lauková et al. (2015). In this study, *Enterococcus faecium* AL41 (10⁹ CFU/ml) was administered at a dose of 400 μl per animal per day in drinking water for 21 days to an experimental group of 40 ostriches. Compared to the control group of 46 ostriches, the treated group showed significant reductions in coagulase-positive and *negative staphylococci, coliforms, Enterobacteria*, and *Pseudomon*as-like bacteria. These findings highlight the antimicrobial potential of *E. faecium* AL41 in improving gut health and managing intestinal microbiota in ostriches. Although limited, these studies indicate that ostrich production could benefit from the application of eubiotics, including probiotics, synbiotics, essential oils, and organic acids, either individually or in combination. Further research is needed to explore their full potential in optimizing growth, immunity, and overall health in ostriches.

### The use of eubiotics in guinea fowl

Guinea fowl is native to Africa, are also commercially reared in Asia, Latin America, and Europe, mainly on the free-range system and on a small scale for meat, eggs, and cash. Their production is saddled with poor hatchability (Sarfo et al. 2018). The use of eubiotics in guinea fowl remains limited, highlighting the need for future research to explore the potential benefits of these additives in optimizing production. The high mortality in keets is due to their weak immunity, which makes them highly susceptible to microbial infection. Accordingly, early supplementation with probiotics in keets via feed or drinking water could help bolster immunity and the growth of beneficial bacteria that offer protection against pathogenic microbes. In a study by Sarfo et al. (2018) on indigenous guinea fowls in northern Ghana, direct-fed microbial (DFM) to drinking water at 1.5 ml/L either daily or three consecutive days per week resulted in higher weight gain and lower feed consumption compared to controls, without affecting blood and serum biochemical properties suggesting that DFM supplementation can effectively enhance growth performance in guinea fowls.

A further study by Galosi et al. (2021) supplemented Guinea fowls with a commercial multi-strain probiotic (2 × 10^11 CFU/L), significantly improving intestinal morphology, including increased villus height, width, crypt depth, and goblet cells. This treatment also enriched beneficial cecal microbiota, such as *Oscillospira*, *Eubacterium*, *Prevotella*, and *Ruminococcaceae*, enhancing gut health and resistance against pathogens, with these taxa playing a crucial role in producing short-chain fatty acids beneficial for enterocytes, glucose metabolism, and exhibiting anti-inflammatory effects. Although research on eubiotics in guinea fowl remains scarce, these studies suggest that probiotic supplementation could enhance growth performance, gut morphology, and immune defense in keets. Further investigations are needed to explore the optimal types and dosages of eubiotics for improving guinea fowl production.

### The use of eubiotics in geese

Geese production is gaining prominence especially in integrated livestock-crop production systems. Although not widely reported, the few studies reported on eubiotic use in geese so far show a promising trend (Table 1). A study by Li et al. (2025) tested the effects of *Bacillus subtilis* (C-3102) on geese at doses of 30 and 60 ppm. The 60 ppm resulted higher final body weight and daily gain while both doses improved feed efficiency, ileum villi height, and digestive enzyme activity. The 60 ppm group also exhibited better nutrient digestibility and increased beneficial gut bacteria. Overall, 60 ppm *B. subtilis* supplementation enhanced growth, digestion, and gut health in geese (Li et al. 2025). Other studies using *B. subtilis* have been reported to improve hatching rate, eggshell thickness, antioxidant capacity, intestinal structure, and beneficial gut microbiota (Wang et al. 2020; Fan et al. 2022).

## Limitations on the use of eubiotics in non-chicken poultry species

The use of eubiotics in poultry production has shown great promise, but it is contingent upon navigating a range of multifaceted challenges. These challenges encompass diverse dimensions, including the need for consistent and reliable efficacy across different poultry species and production systems. Achieving this requires a nuanced understanding of the interplay between eubiotics, gut microbiota composition, host genetics, and environmental factors (Spurgeon et al. 2020; Mallott and Amato 2021). Tailored eubiotic dietary formulations, essential for optimizing benefits in poultry production, must consider species-specific responses influenced by physiological and metabolic disparities among poultry species, encompassing farm specificity. Literature reveals that the efficacy of some eubiotic additives varies across poultry species, as shown in Table 1, necessitating a tailored approach to maximize benefits. Balancing appropriate eubiotic dosage and administration methods is vital, as suboptimal dosing can affect outcomes and production costs, with gut microbiota stability being a critical concern for poultry health and performance (Cao et al. 2021). Eubiotics’ long-term impact on gut microbiota stability necessitates further investigation to prevent adverse effects on host health and productivity, while their incorporation as feed additives faces regulatory and market complexities compounded by interactions with various other additives. Untangling these interactions is crucial for optimizing feed formulations and production outcomes. Poultry producers must assess the economic implications of using eubiotics compared to traditional additives. Despite increasing interest, significant gaps in understanding eubiotics’ mechanisms, optimal usage, and long-term effects persist.

Moreover, immunological differences between conventional and emerging poultry species might influence how eubiotics modulate their immune systems. Therefore, deciphering the precise immunomodulatory mechanisms of eubiotics in quail, turkey, ostrich, ducks, and guinea fowl is paramount to harnessing their full potential in improving disease resistance. Further, dosing recommendations for eubiotics often come from suppliers driven by vital commercial interests, casting doubt on these guidelines’ validity and scientific rigour (Aruwa et al. 2021). There is a pressing need for more robust, independent research to establish appropriate dosages for diverse poultry species, ensuring both effectiveness and cost-efficiency. Another critical issue is the lack of standardized assay methods to accurately assess the active molecules within eubiotics. In contrast to feed enzymes, many eubiotics lack reliable measurement techniques. Consequently, producers and researchers face difficulties in determining precise additive levels in feed, hampering the interpretation of animal responses, which leads to inconsistent or inconclusive trial outcomes.

Moreover, while commercial poultry diets routinely combine multiple feed additives, much of the published research predominantly focuses on the effects of individual components in controlled settings. This knowledge gap creates a disparity between the experiences of researchers and farmers employing complex combinations of eubiotics and other supplements (Bean-Hodgins and Kiarie 2021). A deeper understanding of potential additive and synergistic interactions within mixed diets is crucial for practical and effective application.

## Conclusion and prospects

Eubiotics hold immense promise for NCP production, with potential benefits ranging from enhanced gastrointestinal health to improved product quality as shown in this review. This review showed that applying eubiotics produces different results depending on the species type, dosage level, feeding approach, and rearing conditions. However, tackling the myriad challenges of efficacy, species-specific responses, administration, gut microbiota stability, regulations, interactions, costs, and knowledge gaps is essential for their successful adoption in NCP production systems. Thus, correct application methods of eubiotics, whether singly or combined, require further research to ensure environmental, social, and economic sustainability of NCP production systems. This approach would ensure these birds continue contributing to global food safety and nutrition security.

## Data Availability

All available data is included in the manuscript.
